# Queckenstedt’s test repurposed for the quantitative assessment of the cerebrospinal fluid pulsatility curve

**DOI:** 10.1007/s00701-023-05583-w

**Published:** 2023-04-20

**Authors:** Najmeh Kheram, Andrea Boraschi, Nikolai Pfender, Andreas Spiegelberg, Vartan Kurtcuoglu, Armin Curt, Martin Schubert, Carl Moritz Zipser

**Affiliations:** 1 Department of Neurology and Neurophysiology, Balgrist University Hospital, Zurich, Switzerland; 2 The Interface Group, Institute of Physiology, University of Zurich, Zurich, Switzerland; 3 Zurich Center for Integrative Human Physiology, University of Zurich, Zurich, Switzerland

**Keywords:** Cerebrospinal fluid pressure, Cerebrospinal fluid, Queckenstedt’s test, Relative pulse pressure coefficient, Spinal cord compression

## Abstract

**Purpose:**

Before the era of spinal imaging, presence of a spinal canal block was tested through gross changes in cerebrospinal fluid pressure (CSFP) provoked by manual compression of the jugular veins (referred to as Queckenstedt's test; QT). Beyond these provoked gross changes, cardiac-driven CSFP peak-to-valley amplitudes (CSFPp) can be recorded during CSFP registration. This is the first study to assess whether the QT can be repurposed to derive descriptors of the CSF pulsatility curve, focusing on feasibility and repeatability.

**Method:**

Lumbar puncture was performed in lateral recumbent position in fourteen elderly patients (59.7±9.3 years, 6F) (NCT02170155) without stenosis of the spinal canal. CSFP was recorded during resting state and QT. A surrogate for the relative pulse pressure coefficient was computed from repeated QTs (i.e., RPPC-Q).

**Results:**

Resting state mean CSFP was 12.3 mmHg (IQR 3.2) and CSFPp was 1.0 mmHg (0.5). Mean CSFP rise during QT was 12.5 mmHg (7.3). CSFPp showed an average 3-fold increase at peak QT compared to the resting state. Median RPPC-Q was 0.18 (0.04). There was no systematic error in the computed metrics between the first and second QT.

**Conclusion:**

This technical note describes a method to reliably derive, beyond gross CSFP increments, metrics related to cardiac-driven amplitudes during QT (i.e., RPPC-Q). A study comparing these metrics as obtained by established procedures (i.e., infusion testing) and by QT is warranted.

## Introduction

An improved assessment of cerebrospinal fluid pressure (CSFP) dynamics may be of great clinical value for the investigation of spinal cord compression and a broad spectrum of CSFP disorders (e.g., syringomyelia, normal pressure hydrocephalus; NPH). In routine clinical practice, CSFP is usually measured with analogue manometers, and opening pressure is determined [36].

Beyond the CSF opening pressure, continuous high-resolution recording enables the measurement of CSFP amplitude variations induced by cardiovascular action (CSFPp). While this has been investigated in selected cohorts of patients suffering from various disorders at bedside (e.g., in intracranial hypertension [5] and Chiari malformation [16]) or intraoperatively (e.g., in syringomyelia [35], spinal cord injury [29], and degenerative cervical myelopathy; DCM [48]), lumbar CSFPp in healthy subjects are rather poorly studied. In 10 patients without intracranial pathology, an average CSFPp of 0.7 mmHg was observed [43], and another study reported an average lumbar CSFPp of 2.0 mmHg in 40 healthy elderly volunteers [25].

The CSFP waveform contains information on the intracranial pressure-volume curve [3]. With rising mean CSFP, CSFPp linearly increases. The slope of such a relationship, quantified through linear regression, is termed relative pulse pressure coefficient (RPPC) and depends on the intracranial elastance as well as on the pulsatile cerebral blood volume change [3]. To prevent errors related to the chosen reference level for the CSFP measurement, a constant P_0_ can be introduced [3]. Theoretically, P_0_ represents the pressure at infinite compliance [25], quantified as the x-axis intercept of the regression line. These metrics can be computed through the infusion of a specific fluid volume into the CSF space while simultaneously recording CSFP. Infusion testing was established in NPH as a safe complementary diagnostic tool [33], with some drawbacks regarding the duration of examination (on the order of at least 1-h overall [1]) and difficulties to assess repeatability [42].

Queckenstedt’s test (QT) is a maneuver originally developed to reveal a spinal cord compression [37]. While it had been employed in diverse patient populations (e.g., DCM [28, 32], tumor-, and infection-related cord compression [27]), it was abandoned in favor of spine imaging due to low sensitivity of CSFP-related metrics. QT is performed by firm manual compression of the jugular veins for 10 s, which increases intracranial venous blood volume by blocking the outflow through the jugular veins. This elevates the mean CSFP, which is related to the venous pressure in the dural sinuses by Davson’s equation [13]. Hence, it reduces intracranial compliance and thereby increases CSFPp. Comprehensive monographies have been written on QT morphology and physiology, for instance by Lakke [30] and Antoni [2], but information on the range for mean CSFP rise during QT are sparse (between 7 and 40 mmHg [2, 20, 41]), and the evolution of CSFPp from resting state to the plateau phase of QT has not systematically been described yet in spine-healthy patients. To our knowledge, only Gilland et al. [21] reported the CSFPp (6 mmHg) during the plateau phase of QT (i.e., at a mean CSFP of 29 mmHg), as observed in 31 healthy volunteers.

We hypothesize that QT can be employed to non-invasively and repeatedly probe the linear relation between mean CSFP and CSFPp. Similar to infusion testing, QT induces a volume challenge for the craniospinal compartment. Through this novel application of QT, potential surrogates for RPPC and P_0_—metrics that are normally obtained by infusion testing—can be calculated. This study aims at providing a range of values for CSFP-derived parameters in a spine-healthy cohort, assessing their repeatability, and comparing the findings to gold standard infusion-based values from the literature. To our knowledge, this is the first study where QT is repurposed to derive CSF pulsatility curve.

## Methods

### Data acquisition

The data was prospectively acquired in patients undergoing lumbar puncture (LP) at our outpatient clinic between December 2019 and April 2021 for reasons other than spinal cord compression, disturbed CSF circulation, or abnormal intracranial pressure (e.g., not including patients with history of NPH). These patients serve as controls within a study investigating CSFP-related metrics in patients with degenerative cervical myelopathy undergoing surgical decompression of the spinal CORD (COMP-CORD) [49] (NCT02170155). This study conformed to the latest revision of the Declaration of Helsinki and was approved by the local Ethics Committee of the University Hospital of Zurich (KEK-ZH number PB-2016-00623).

LP was performed in awake patients with a 21G × 90 mm Sprotte^®^ spinal needle (Pajunk, Geisingen, Germany) or with a 22G Quincke spinal needle (Temena Group, Carrières-sur-Seine, France) in lateral recumbent position. The needle was connected to a pressure transducer (VentrEX^®^ system, Neuromedex GmbH, Hamburg, Germany), which in turn was connected to an interface monitor (Philips X2-Pat. Interface+MX 700, Philips, Amsterdam, the Netherlands) through which the CSFP signal was transmitted to a laptop for recording with ICM+ software (Cambridge Enterprise, Cambridge, UK).

**Fig. 1:**
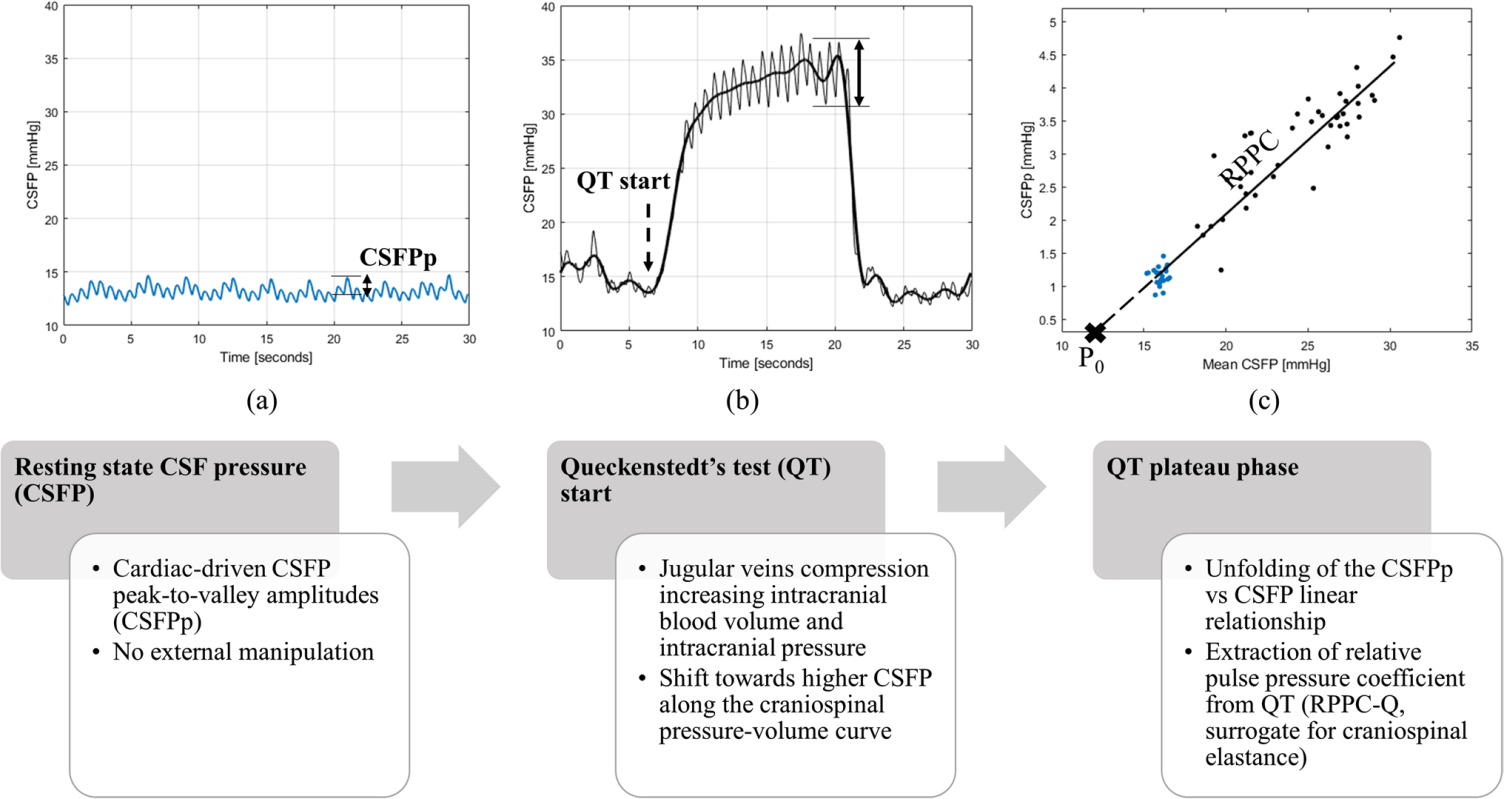
Basic concepts of the study. Flow diagram of cerebrospinal fluid pressure (CSFP) assessment during resting state (**a**), and Queckenstedt’s test (QT) (**b**), and the resulting pulsatility curve (**c**) in a representative patient (ID 059)

An overview of the basic concepts of the study is provided in Fig. 1. First, CSFPp was recorded in the resting state (Fig. 1a). Subsequently, QT was performed (Fig. 1b), twice to assess repeatability. For QT, firm bilateral manual pressure was applied on the neck for 10 s by the same trained investigator throughout the study (CZ). Electrocardiogram (ECG) was recorded in 9/14 patients. The data that support the findings of this study are available from the corresponding author upon reasonable request.

### Calibration of the pressure measurement system

If the pressure transducer is kept at the same hydrostatic level of the needle tip within the lumbar CSF space, it can be assumed that the static value of the actual CSFP and that of the measured pressure signal are equal. However, the dynamic component of CSFP (i.e., CSFPp) will be damped by the elements between the needle tip and the pressure transducer, such as the needle itself and the connecting tubes, due to their geometric and elastic properties.

**Fig. 2:**
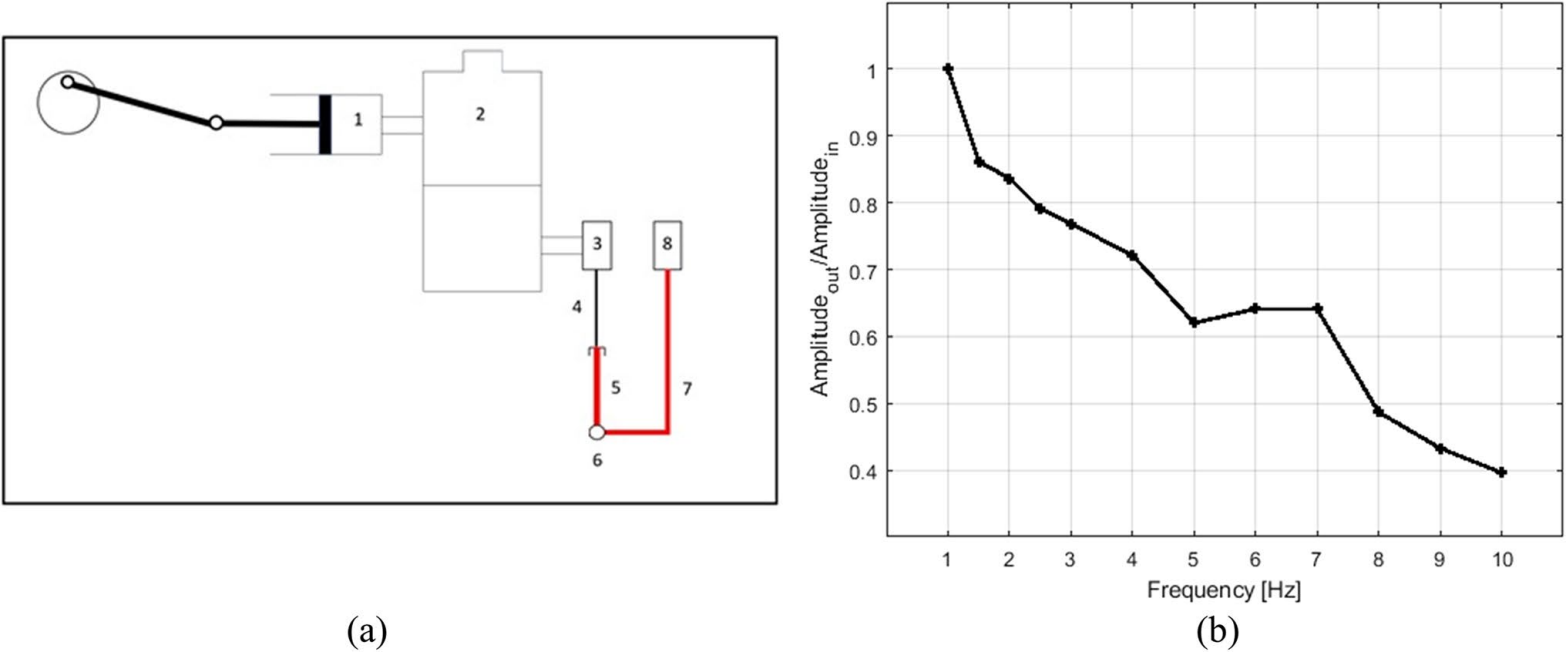
(**a**) Schematic representation of the experimental setup (1–3) employed to test the frequency response of the pressure measurement system (4–8); 1: motor-driven piston pump, 2: bottle semi-filled with water, 3: first pressure transducer, 4: needle, 5: short connecting line, 6: three-way stopcock, 7: connecting tube, 8: second pressure transducer. (**b**) The ratio between the peak-to-valley amplitudes of the output and the input pressure signals, as measured by the second (8) and first pressure transducer (3), respectively, is reported for each investigated frequency

The pressure measurement system, in particular the components between the needle tip and the pressure transducer, was tested with the experimental setup schematically reproduced in Fig. 2a. The components used for the pressure measurement system belong to the VentrEX^®^ system. Pressure transmission was investigated by varying the frequency of the generated sinusoidal pressure wave in the range between 1 and 10 Hz, with a step of 0.5 Hz between 1 and 3 Hz (the most interesting range for cardiac modulation) and of 1 Hz between 3 and 10 Hz.

### Cerebrospinal fluid pressure metrics

**Fig. 3:**
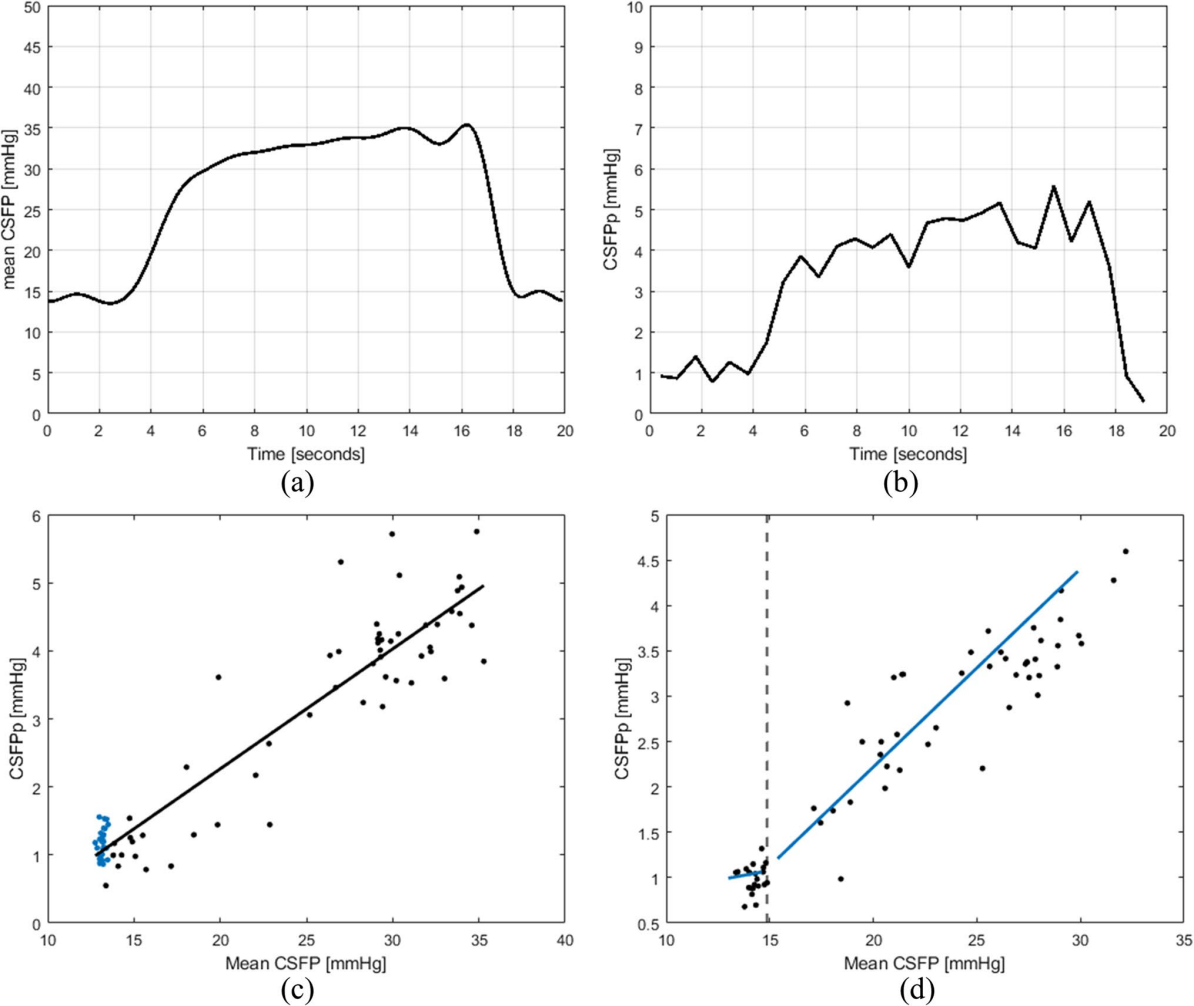
Queckenstedt’s test (QT) derived cerebrospinal fluid pressure (CSFP) parameters. (**a**) Mean CSFP calculated using the time-reconstructed CSFP signal obtained with the frequency bins in the range 0–0.5 Hz. (**b**) Cardiac-driven CSFP peak-to-valley amplitude (CSFPp) followed from resting state and to QT, calculated using the time-reconstructed CSFP signal obtained with the frequency bins in the range 0.5–8 Hz. (**c**) CSFPp at resting state (blue dots) and during QT (black dots) is plotted against mean CSFP. The regression line (in black) is reported: the relative pulse pressure coefficient (RPPC-Q, 0.18) corresponds to the slope of such a line and P_0_-Q (7.15) to the x-axis intercept. (**d**) Data from a single patient (ID 053) showing CSFPp at resting state (left of the vertical dashed line) and during QT (right of the vertical dashed line); during QT the positive correlation between mean CSFP and CSFPp is detected (*P* < 0.001, correlation coefficient = 0.66)

For each patient’s dataset, three time windows were selected from the whole recording: one in the resting state and two containing a QT each, all 20 s long. Discrete wavelet decomposition was applied to the CSFP signal with Daubechies mother wavelets [12]. CSFP signal was, in this way, decomposed into different frequency bins, with lower bins containing low-frequency ranges (e.g., mean CSFP and breathing modulation) and higher bins including higher-frequency content (e.g., pulsations from cardiovascular action and their harmonics). The data in each frequency bin contributed to the time-domain reconstruction of the CSFP signal. Mean CSFP was computed as the sum of the first four frequency bins (0–0.5 Hz, Fig. 3a). The signal reconstructed using the sum of the next four frequency bins (0.5–8 Hz) was used for the calculation of CSFPp (Fig. 3b). CSFPp corresponds to the difference between the systolic peak and the associated diastolic valley. To quantify RPPC-Q and P_0_-Q, a line was fitted using linear regression to the CSFPp vs. mean CSFP from individual subject’s data points of the resting state and QT sections, and its slope and intercept were extracted (Fig. 3c). RPPC-Q corresponds to the slope of such a regression line and P_0_-Q to the x-axis intercept. Unless differently specified, the median (interquartile range) is reported for the investigated metrics.

Bland-Altman plots were computed to investigate the repeatability of QT, by analyzing the repeatability coefficient between the two maneuvers performed in each patient. Four QT-derived metrics were considered: maximum CSFP rise during QT, CSFPp at peak QT, RPPC-Q, and P_0_-Q. The bias, i.e., average of the differences between the two QTs, is provided along with its 95% confidence interval. The repeatability coefficient is set at ± 1.96*s* from the bias [34], where *s* is the standard deviation of the differences between the two QTs.

## Results

### Demographics and clinical characteristics

Fourteen patients (59.7±9.3 years, 6 females) underwent routine diagnostic LP for suspicion of autoimmune demyelinating central nervous system (CNS) disease (*N*=7), inflammatory peripheral neuropathy (*N*=4), or infectious CNS disease (*N*=3). All patients were in stable medical conditions, and LP was performed electively (outpatient setting). None of the patients had undergone LP previously. Cervical magnetic resonance imaging (MRI) scans were acquired in most patients (11/14, 79%) for reasons other than suspicion of cord compression. No adverse events related to LP or to the CSFP provocation maneuvers were reported. Patient characteristics are provided in Table 1.

**Table 1:** Patients’ characteristics

ID	Age [years]	Weight [kg]	Height [cm]	BMI [kg/m^2^]
050	50	51	166	18
052	39	60	160	23
053	65	72	175	23
054	53	75	182	23
055	72	70	153	30
058	62	77	180	24
059	71	87	172	29
060	60	86	173	29
061	73	75	167	27
062	60	85	175	28
063	55	65	168	23
064	65	74	180	23
065	50	68	156	28
066	61	85	192	23

### Calibration of the pressure measurement system

Figure 2b shows the performance of the employed pressure measurement system in the frequency range from 1 to 10 Hz, reporting the ratio between the peak-to-valley amplitudes of the output and the input pressure signals, as measured by the second (indicated by number 8 in Fig. 2a) and the first pressure transducer (indicated by number 3 in Fig. 2a), respectively. At 1 Hz, this ratio is equal to 1, whereas at all the other investigated frequencies a certain damping is observed. In particular, the setup exhibits a linear response, interrupted from 5 to 7 Hz possibly by resonance related phenomena. However, considering that more than 90% of the power in the CSFP signal related to cardiovascular action is contained below 2 Hz, the output to input ratio shown by the pressure measurement system was considered acceptable for the frequency range of interest.

### Cerebrospinal fluid pressure metrics

Mean CSFP in the resting state was 12.3 (IQR 3.2) mmHg with CSFPp of 1.0 (0.5) mmHg. CSFP rise during QT was 14.3 (6.8) mmHg for the first test, without significant difference compared to the second test performed (11.3 [8.2] mmHg, *P* = 0.63). CSFPp at peak QT was 3.7 (1.9) mmHg, showing a 3-fold increase compared to the resting state (*P* < 0.0001). Detailed values for individual subjects’ CSFP parameters are provided in Table 2.

**Table 2:** Cerebrospinal fluid pressure (CSFP) data for all patients at resting state, and during the two Queckenstedt’s test (QTs)

ID	Mean CSFP^*^	CSFPp^*^	△CSFP^*^	CSFPp^*+^	△CSFP^*^	CSFPp^*+^	RPPC-Q	P_0_-Q^*^
050	16.6 (0.5)	0.5 (0.2)	9.0	1.8	9.4	2.4	0.22	14.2
052	15.1 (0.4)	1.2 (0.2)	9.9	2.9	6.4	2.7	0.17	8.4
053	14.3 (0.6)	1.0 (0.2)	15.1	4.2	9.2	3.6	0.18	8.3
054	10.1 (0.4)	0.4 (0.1)	11.2	2.6	5.3	1.5	0.15	8.1
055	12.4 (0.5)	2.1 (0.4)	16.9	7.6	10.8	7.7	0.44	6.3
058	9.8 (0.5)	1.2 (0.2)	9.8	2.2	11.3	5.1	0.20	3.5
059	13.1 (0.5)	1.2 (0.3)	15.6	4.4	21.9	5.1	0.18	7.1
060	8.2 (0.3)	1.0 (0.2)	10.5	3.1	14.5	3.7	0.13	-0.1
061	11.1 (0.2)	0.9 (0.2)	13.7	2.8	11.3	3.0	0.13	2.6
062	12.3 (0.3)	0.7 (0.1)	22.4	4.6	21.2	4.6	0.19	8.2
063	11.1 (0.6)	0.7 (0.3)	14.9	4.0	6.3	3.8	0.18	7.1
064	13.2 (0.4)	1.0 (0.3)	10.1	2.9	17.4	4.3	0.17	7.5
065	12.2 (0.4)	1.4 (0.3)	27.6	6.7	34.1	8.8	0.21	6.1
066	18.9 (0.5)	0.4 (0.1)	19.9	2.7	16.3	2.6	0.10	13.3

Mean CSF pressure (mean CSFP), cardiac-induced CSF pressure peak-to-valley amplitude (CSFPp), mean CSFP rise (∆CSFP), relative pulse pressure coefficient obtained through QT (RPPC-Q), pressure at infinite compliance obtained through QT (P_0_-Q). At resting state (median [interquartile range])

*In mmHg

+Median of the five highest values

**Fig. 4:**
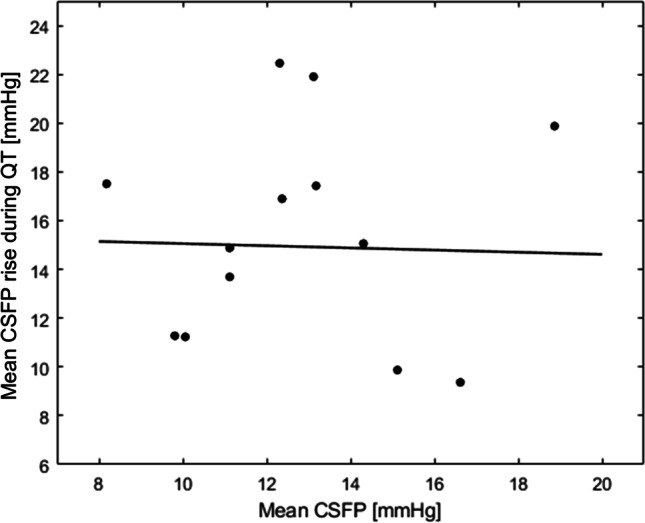
Cerebrospinal fluid pressure (CSFP) rise plotted against mean CSFP at the resting state (no significant correlation, *P* = 0.867); each dot represents a patient (*n* = 14)

Figure 4 shows CSFP rise during QT plotted against mean CSFP in the resting state, with subject-by-subject data. Using Kendall correlation coefficient (*τ*), since both variables were not normally distributed, a weak correlation was observed (*τ*=0.10). The relationship between CSFPp and the corresponding mean CSFP provides information about the craniospinal pressure-volume curve. A linear relationship exists between CSFPp and the corresponding mean CSFP, with higher mean CSFP showing higher CSFPp [3]. However, as shown in Fig. 3d, the range of mean CSFP observed in the resting state fails to illustrate this physiological correlation. Conversely, QT allows investigating a broader range of mean CSFP, permitting detection of the positive correlation between CSFPp and the corresponding mean CSFP (*τ*=0.66, *P* < 0.0001, Fig. 3d).

Figure 3c shows the relationship between CSFPp and mean CSFP at resting state and during QT for a single patient (ID 053). The parameters extracted from the calculated regression line are the slope, considered a surrogate of the relative pulse pressure coefficient (RPPC-Q), and the x-axis intercept, considered a surrogate of the theoretical pressure at infinite compliance (P_0_-Q). RPPC-Q and P_0_-Q were quantified for the whole patient cohort as 0.18 (0.04) and 7.3 (2.20) mmHg. Detailed individual subjects’ values are provided in Table 2.

**Fig. 5:**
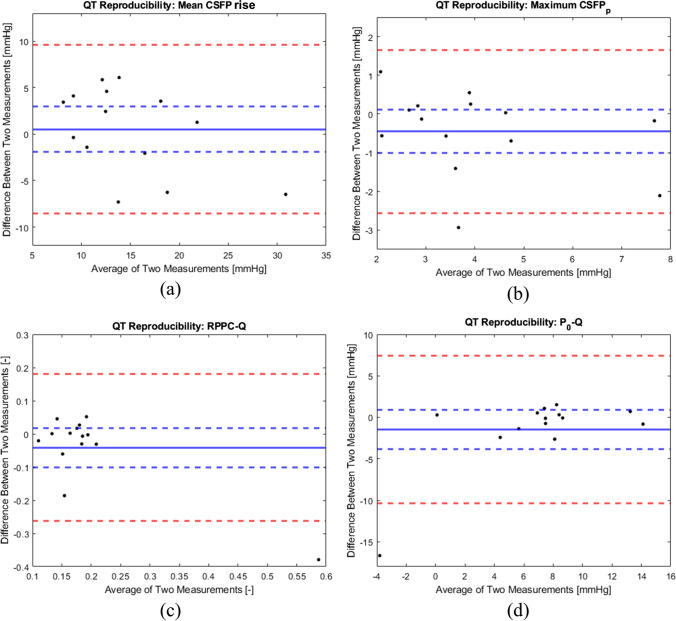
Bland-Altman plots of Queckenstedt’s test (QT) derived metrics. Each dot represents single patient’s data, since two QTs were performed in each patient. (**a**) Cerebrospinal fluid pressure (CSFP) rise; bias: 0.8 mmHg (continuous blue line), 95% confidence interval of the bias: −1.9 – 3.6 mmHg (dashed blue lines), repeatability coefficient: −9.5 – 11.1 mmHg (dashed red lines). (**b**) Cardiac-driven CSFP peak-to-valley amplitude (CSFPp); bias: −0.5 mmHg (continuous blue line); 95% confidence interval of the bias: −1.0 – 0.1 mmHg (dashed blue lines); repeatability coefficient: −2.6 – 1.6 mmHg (dashed red lines). (**c**) Relative pulse pressure coefficient obtained through QT (RPPC-Q); bias: −0.04 (continuous blue line), 95% confidence interval of the bias: −0.10 – 0.02 (dashed blue lines), repeatability coefficient: −0.26 – 0.18 (dashed red lines). (**d**) Theoretical pressure at infinite compliance obtained through QT (P_0_-Q); bias: −1.5 mmHg (continuous blue line), 95% confidence interval of the bias: −3.8 – 0.9 mmHg (dashed blue lines), repeatability coefficient: −10.3 – 7.4 mmHg (dashed red lines)

Figure 5 shows the Bland-Altman plots of QT-derived metrics. The maximum CSFP rise during QT showed a bias of 0.8 mmHg, with repeatability coefficient of −9.5 and 11.1 mmHg, respectively. The CSFPp at peak QT had a bias of 0.5 mmHg, with repeatability coefficient of −2.6 and 1.7 mmHg, respectively. RPPC-Q showed a bias of −0.04, with repeatability coefficient of −0.26 and 0.18, respectively. P_0_-Q had a bias of −1.5 mmHg, with repeatability coefficient of −10.4 and 7.4 mmHg, respectively. In none of the reported Bland-Altman plots an association between the difference and the average of the two measurements was observed. In all the reported Bland-Altman plots the 95% confidence interval of the bias includes 0, indicating that there is no systematic error between the first and the second measurement.

## Discussion

### Summary of main findings

This study provides a range for mean CSFP and CSFPp in the resting state and during QT in a cohort of elderly patients without spinal cord compression. QT allows varying the mean CSFP in a reproducible way and to an extent that unfolds the linear relationship between mean CSFP and CSFPp, which cannot be detected by using the resting state CSFPp. Thereby, QT was employed as an auto-infusion test to derive surrogate metrics for the relative pulse pressure coefficient (RPPC-Q) and for the theoretical pressure at infinite compliance (P_0_-Q). This novel approach represents a significant advance from the standard QT analysis, which focused on CSFP rise from baseline.

### Queckenstedt’s test: physiology and comparison to the literature

QT can be viewed as a maneuver challenging the craniospinal compliance, since intracranial blood volume increases due to blockage of outflow through the jugular veins. Because of the filling of intracranial veins, the pressure in the dural venous sinuses rises, resulting in an elevation of CSFP [13]. At the same time, while arterial blood supply into the craniospinal compartment is maintained to guarantee adequate perfusion of the central nervous system, the compliance of the compartment is reduced due to its higher filling state, which translates into a higher CSFPp. In patients without spinal cord compression, CSFP dynamics can be reliably measured at lumbar level due to the communicating craniospinal CSF space [4, 6, 31]. In one of the largest published cohort series, Stookey and colleagues [41] reported a physiological maximum CSFP increase of 7 – 15 mmHg in 125 patients with no suspicion of spinal block from the CSFP examination, resembling the values we obtained (14.3 [6.8] mmHg, with some high outliers). This comparison illustrates that the value of CSFP rise during QT has promising intertrial repeatability, but also that large QT ranges render individual decision making difficult. Importantly, our results indicate that the degree of responsiveness to QT (i.e., the highest CSFP reached during the plateau phase of the maneuver) in spine-healthy patients is not influenced by the mean CSFP in the resting state.

### Exploring QT metrics: higher repeatability and improved sensitivity for spine disease?

One cause for the drop of interest in QT was its low to moderate sensitivity to detect spinal cord compression based solely on absolute values of mean CSFP rise from the resting state to peak QT [15, 45, 47]. In a cohort of patients with chronic spinal canal narrowing, we found that RPPC-Q was more sensitive to detect effective stenosis than rise of CSFP during Queckenstedt's test [28]. Moreover, QT is performed manually and might be affected by various factors (e.g., how the venous outflow in the other minor veins of the neck adapts to the collapse of jugular veins [9]). Therefore, we explored more metrics derived from QT and investigated their repeatability through Bland-Altman plots by comparing results obtained in two subsequently performed QT. We observed that in all the plots, the confidence interval of the bias included zero, indicating the lack of a systematic error between the two maneuvers. For the maximum CSFP rise during QT, less than half of the data points lie within the 95% confidence interval of the bias, having a width of 5.5 mmHg (Fig. 5a). Conversely, for P_0_-Q, despite a smaller width (4.7 mmHg) compared to that of CSFP rise, almost 80% of the data points lie within the 95% confidence interval of the bias (Fig. 5d). By considering a clinically relevant interval of ±1 mmHg for the difference between two measurements of CSFPp, more than 70% of the data points lie within (Fig. 5b). Based on the values reported in the literature for RPPC and on their high variability [17, 25, 43], the fact that most of the data points (85%) lie in the ±0.1 interval (Fig. 5c) was considered as a good indication for this metric’s repeatability. Indeed, comparing our Bland-Altman plot for RPPC-Q to that reported in [42], where the repeatability of infusion testing was assessed, we observed similar bias and repeatability coefficients. It should be noted that the reported 95% confidence interval of the bias does not have any clinical significance per se. Indeed, considerations based on literature and clinical experience are required to assume that the range of a metric is deemed acceptable for repeatability [19]. Based on the four Bland-Altman plots, we conclude that RPPC-Q and P_0_-Q are more repeatable than CSFP rise and CSFPp at peak QT.

### CSFP, CSFPp, and proxies for RPPC and P_0_: comparison to the literature

The measured range for mean CSFP in the resting state (8.6 to 18.9 mmHg) matches well with the range reported in a recent systematic review (7.2 to 16.8 mmHg [36]). Similarly, the findings on CSFPp (0.4 to 2.2 mmHg) align with the values present in the literature (0.7 to 3.7 mmHg [21, 25, 43]).

Even though infusion testing represents an established procedure in the diagnosis and outcome prediction of CSFP disorders [8, 26], some heterogeneity exists among the reported values of RPPC and P_0_ determined through such testing. In healthy subjects, RPPC was found to be 0.19±0.06 [17], 0.33±0.08 [43], and 0.62±0.23 [25]. Fewer studies investigated P_0_ in a healthy cohort. Jacobsson and colleagues reported a value of 9.1±2.7 mmHg [25]. RPPC is related to the elastance coefficient *E* [3], a parameter that defines the slope of the craniospinal pressure-volume curve, and to the pulsatile cerebral blood volume change. A value of *E* higher than 0.18 ml^−1^ has been considered an indicator of disturbed CSF compensatory reserve [11], whereas a value equal to 0.1 ml^−1^ as physiological [18, 39]. Assuming a pulsatile cerebral blood volume change of 2 ml [25], the median elastance coefficient in our whole cohort was 0.08 (0.02) ml^−1^, thus in accordance with the reported values for healthy subjects. However, such calculation needs to be cautiously considered, since the pulsatile cerebral blood volume change has a high inter-patient variability (0.36 to 4.38 ml, according to [3]). Thus, our estimation of *E* is purely indicative.

The obtained values of RPPC-Q and P_0_-Q (0.18 [0.04] and 7.3 [2.20] mmHg) align well with the infusion-derived values of RPPC and P_0_ reported in [17]. At the same time, the obtained values of RPPC-Q and P_0_-Q underestimate the values of RPPC and P_0_ reported in [42, 24]. A possible explanation for this would be that such a comparison needs caution, as the two underlying procedures modulate CSF circulation in different ways. Infusion testing consists of direct addition of volume into the CSF spaces, determining a rise in the mean CSFP with a concomitant increase of CSFPp due to reduced craniospinal compliance. Contrariwise, given that in the constant flow infusion test the infusion rate is on the order of few ml/min, the craniospinal compartment has more time to adapt than during QT as increased CSF outflow occurs until a plateau mean CSFP is reached and a new equilibrium working point is established. In this regard, the infusion test not only serves to investigate the biomechanical properties of a stable system but also provokes physiological adaptations by permanently increasing the filling state and thus offsetting balance within available CSF space. In contrast, QT acts indirectly on the CSF space within a short timeframe (seconds), since it provokes a similar response of the CSFP signal by temporarily increasing intracranial volume following blocked outflow of venous blood through the jugular veins. Indeed, not only the mean CSFP increases due to its relationship with the venous pressure in the dural sinuses, but also CSFPp rises because of the reduced compliance of the craniospinal space.

### Clinical implications

Advanced QT analysis potentially expands its utility in the investigation of syringomyelia [44], DCM [49], and headache disorders [10, 14, 24]. In the field of communicating CSFP disorders [16, 23], clinicians may refer to the CSFPp range from this healthy cohort when evaluating CSFP dynamics. In addition, the use of QT to derive CSF pulsatility curve may be of interest for specialists using infusion testing. The potential advantages of QT are its short examination time and no requirement for additional devices. As such, it may add complimentary information to a comprehensive diagnostic workup including clinical and imaging parameters, and a full range CSFP dynamics evaluation. It could be valuable particularly in patients who do not tolerate longer investigation times as needed during infusion testing. The repeatability of RPPC-Q and P_0_-Q in our cohort warrants applicability of this method, which needs to be further explored with respect to responsiveness, e.g., in cohorts of patients suffering from various CSFP disorders. Of note, we found some damping of CSFPp related to the pressure measurement system. However, such damping was minor and thus deemed acceptable in the frequency range of interest. The quantification of this aspect is often neglected in studies investigating the CSFP signal, and it should be considered when comparing the presented findings to the literature. Importantly, advanced QT is not a substitute for neuroimaging and neurological examinations but may provide valuable additional information for clinical decision making.

It is important to note that the clinical utility of the CSF pulsatility curve could be argued even when derived with infusion testing. Indeed, there is a lack of agreement in the literature about this. While Jacobsson and colleagues observed a shift of such a curve in hydrocephalic patients compared to healthy controls, van Bilsen and colleagues [7] did not observe any change of the curve after shunt surgery. In this regard, we would like to underline the high failure rate (30%) in computing CSF pulsatility curve, which according to the author themselves is an aspect warranting for further studies. Furthermore, in another study by our group in patients with DCM, the acquired CSF metrics showed a clear distinction between a blocked and a healthy spinal canal and proved as a useful tool alongside conventional diagnostic methods such as neurophysiological tests and MRI. Thus, in patients with spinal canal encroachment and mutual spinal cord compression, advanced QT analysis holds potential to determine the extent of spinal cord obstruction.

### Limitations

Despite the novelty and several strengths of our study, there are some limitations to point out. First, we provided CSFP-related values from patients with neurological diseases other than spinal cord compression, such as multiple sclerosis, but not from healthy controls. However, the clinical conditions of our subjects are not considered to have a significant impact on CSFP dynamics. Secondly, due to the limited size of the investigated cohort and the narrow range of patients’ characteristics (i.e., age, height, weight, BMI), we deemed a correlation analysis between such characteristics and QT metrics not informative. Moreover, the values we found are valid for elderly patients, whereas younger adults may behave differently. Thirdly, we investigated repeatability of QT within one LP, but not intertrial repeatability across different LP sessions, which might be needed for follow-up investigations. This is a difficult endeavor, though, because the CSFP environment is a dynamic system. For instance, changes in volume status and body position may already change the response to manual pressure [40]. In addition, although QT was performed by the same investigator throughout the trial, it cannot be fully standardized. In a clinical setting we would not consider more standardized methods feasible, e.g., to perform QT with an inflatable neck cuff [22]. In practice, monitor-controlled QT may deliver better test-retest results. Its utility to derive surrogates of RPPC and P_0_ might be explored best in patients with CSFP disorders undergoing the gold standard of infusion testing and QT as a comparator. Furthermore, no volume measurement was performed during QT, which represents a limitation compared to infusion test, where the volume of artificial CSF injected into the CSF system is known. Finally, for intraoperative monitoring of spinal cord decompression, intraspinal pressure monitoring has been recently pioneered to provide information on the pressure conditions at the level of lesion [38, 46]. The ranges reported here do not necessarily correspond to intraspinal pressure as they represent a different compartment.

## Conclusions

In this cohort of spine-healthy patients, the Queckenstedt’s test (QT) determined a 2-fold increase of mean cerebrospinal fluid pressure (CSFP), and a 3-fold rise in cardiac-driven peak-to-valley CSFP amplitude (CSFPp). In this technical note we described a simplified method to derive surrogates for the relative pulse pressure coefficient (RPPC-Q) and pressure at infinite compliance (P_0_-Q) from CSFP tracing during QT. The applicability of these metrics in the context of CSFP disorders must be empirically tested by clinical studies comparing QT assessment with the established methods (i.e., infusion testing).

## Abbreviations

CNS: Central nervous system
CSF: Cerebrospinal fluid
CSFP: Cerebrospinal fluid pressure
CSFPp: Cardiac-driven CSFP peak-to-valley amplitude
DCM: Degenerative cervical myelopathy
E: Elastance coefficient
ECG: Electrocardiogram
LP: Lumbar puncture
MRI: Magnetic resonance imaging
NPH: Normal pressure hydrocephalus
P_0_: Pressure at infinite compliance
P_0_-Q: Pressure at infinite compliance obtained using QT
QT: Queckenstedt’s test
RPPC: Relative pulse pressure coefficient
RPPC-Q: Relative pulse pressure coefficient obtained using QT
